# How to Build a Patient-Specific Hybrid Simulator for Orthopaedic Open Surgery: Benefits and Limits of Mixed-Reality Using the Microsoft HoloLens

**DOI:** 10.1155/2018/5435097

**Published:** 2018-11-01

**Authors:** Sara Condino, Giuseppe Turini, Paolo D. Parchi, Rosanna M. Viglialoro, Nicola Piolanti, Marco Gesi, Mauro Ferrari, Vincenzo Ferrari

**Affiliations:** ^1^EndoCAS Center, Department of Translational Research and of New Surgical and Medical Technologies, University of Pisa, Pisa, PI 56124, Italy; ^2^Department of Information Engineering, University of Pisa, Pisa, PI 56124, Italy; ^3^Department of Computer Science Department, Kettering University, Flint, MI 48504, USA; ^4^1^st^ Orthopaedic and Traumatology Division, Department of Translational Research and of New Surgical and Medical Technologies, University of Pisa, Pisa, PI 56124, Italy; ^5^Department of Translational Research and of New Surgical and Medical Technologies, University of Pisa, Pisa, PI 56124, Italy; ^6^Center for Rehabilitative Medicine “Sport and Anatomy”, University of Pisa, Pisa, PI 56124, Italy

## Abstract

Orthopaedic simulators are popular in innovative surgical training programs, where trainees gain procedural experience in a safe and controlled environment. Recent studies suggest that an ideal simulator should combine haptic, visual, and audio technology to create an immersive training environment. This article explores the potentialities of mixed-reality using the HoloLens to develop a hybrid training system for orthopaedic open surgery. Hip arthroplasty, one of the most common orthopaedic procedures, was chosen as a benchmark to evaluate the proposed system. Patient-specific anatomical 3D models were extracted from a patient computed tomography to implement the virtual content and to fabricate the physical components of the simulator. Rapid prototyping was used to create synthetic bones. The Vuforia SDK was utilized to register virtual and physical contents. The Unity3D game engine was employed to develop the software allowing interactions with the virtual content using head movements, gestures, and voice commands. Quantitative tests were performed to estimate the accuracy of the system by evaluating the perceived position of augmented reality targets. Mean and maximum errors matched the requirements of the target application. Qualitative tests were carried out to evaluate workload and usability of the HoloLens for our orthopaedic simulator, considering visual and audio perception and interaction and ergonomics issues. The perceived overall workload was low, and the self-assessed performance was considered satisfactory. Visual and audio perception and gesture and voice interactions obtained a positive feedback. Postural discomfort and visual fatigue obtained a nonnegative evaluation for a simulation session of 40 minutes. These results encourage using mixed-reality to implement a hybrid simulator for orthopaedic open surgery. An optimal design of the simulation tasks and equipment setup is required to minimize the user discomfort. Future works will include Face Validity, Content Validity, and Construct Validity to complete the assessment of the hip arthroplasty simulator.

## 1. Introduction

Surgical simulation, a key enabling technique to revolutionize patient care and patient safety, can provide a standardized method for surgical training without the risks that come with operating on real patients [1].

Orthopaedic simulation has generally lagged behind other specialties, with fewer validated simulators available; this trend is now changing and recent studies support the notion that orthopaedic simulators have the potential to translate useful technical skills into the operating theatre [2].

Several techniques of simulation are available today, including virtual reality (VR) simulation, physical simulation, and hybrid (virtual-physical) simulation.

Existing VR orthopaedic simulators are limited by a poor haptic feedback. One of the major issues to be addressed is the simplification of the computational models to speed up the interactive simulation without compromising the effective realism of the tissue response [3]. Moreover, conventional haptic interfaces are limited in the magnitude of the forces being rendered, so they do not enable a realistic simulation of the surgical instruments/bone interaction, particularly in open surgery where the interaction forces can be of considerable magnitudes. This could explain why, in a recent study [2], Morgan et al. found that commercially available VR simulators are mainly focused on arthroscopy, a minimally invasive procedure.

As for physical simulation, companies like Sawbones [4] offer orthopaedic training models for open surgery procedures such as joint replacement surgery. The strength of these simulators lies in the realism of the synthetic bone, which requires no special handling or preservation and exhibits mechanical properties similar to human bone [5–7]. This is very important for a good simulation experience to allow the surgeon to develop a force-feedback memory, which is crucial for the success of a surgical procedure including tasks such as bone drilling. However, standard commercial mannequins lack objective assessment of performance and cover a very limited range of individual differences and pathologies. Patient-specific simulation, a new frontier that promises great benefits for surgical training and rehearsal [8–10], can overcome this latter limitation.

As suggested by a literature review on orthopaedic surgery simulation [11], *“an ideal simulator should be multimodal, combining haptic, visual and audio technology to create an immersive training environment.”* Hybrid simulation technologies, which combine VR with physical models of the anatomy, are the best candidate to meet these requirements. Hybrid systems indeed have the advantages of physical simulators, which can mimic the properties of human tissue [12–14] offering the trainee the possibility to use actual surgical instruments and experience a realistic haptic feedback; and, at the same time, they exploit the benefit of computer visualization and simulation, offering also objective tools for assessing the surgical performance. Moreover, augmented reality (AR) elements can be added to enrich the synthetic environment, to make hidden structures visible, and to present additional information for the surgical tasks guidance [10, 15–19]. Finally, spatial sound can be added in AR applications to improve the realism of the simulated scenario.

Available display technologies for AR include spatial displays (screen-based and projection-based); hand-held displays (such as phones and tablets); and head-mounted displays (HMDs). HMDs are deemed as the most ergonomic solution for applications including manual tasks performed by the user under direct vision, like what happens in open surgery. HMDs indeed intrinsically provide the user with an egocentric viewpoint and they allow the user to work handsfree [20].

This work explores the potentialities offered by mixed-reality (MR) using the HoloLens [21], an head-mounted display designed by Microsoft for MR applications, to develop an hybrid training system with immersive and interactive content.

Hip arthroplasty (HA), which involves replacing a damaged hip joint with a prosthetic implant, was chosen as a benchmark to evaluate the benefits/limits of the proposed system because it is one of the most widely performed procedures in orthopaedic practice [22], and there is a gap in the market for a high-fidelity hip replacement training simulators [11].

In a previous work [23], we have presented a lower torso phantom for HA including a patient-specific hemi-pelvis replica embedded in a soft synthetic foam. In this paper, we present the HipSim app: an evolution of our former simulator, focusing on the details for the implementation of wearable AR functionalities using the HoloLens. Quantitative and qualitative test were carried out to perform a preliminary evaluation of our multimodal surgical simulator and to explore advantages and limits of the new design and novel technologies being used.

## 2. Materials and Methods

The following paragraphs describe the peculiarities of the adopted HMD; the virtual content and the physical components of the simulator, with details on the implementation/fabrication strategy; the calibration and registration methods to align the VR content with the physical word; and the testing strategies to preliminary validate the simulator.

### 2.1. Selection of the Head-Mounted Display

HoloLens is an Optical See-Through (OST) HMD, which enables optical superposition of virtual content onto the user direct view of the physical world. Being an OST system, it offers an unhindered and instantaneous full-resolution view of the real environment which assures that visual and proprioception information is synchronized [24].

Differently, in Video See-Through (VST) HMDs, the virtual content is merged with the camera images captured by one or two external cameras rigidly fixed on the visor frame. This more obtrusive technology block out the real-world view in exchange for the ability to offer higher geometric coherence between virtual and real content, without requiring a user-specific calibration eye-to-display [25]. A complete comparison of OST and VST technologies is reported in [26].

Assuming that for simulation purposes the perceived positioning accuracy of the VR content is not as important as the possibility to give the user a naturalistic experience, we have opted for an OST system. More in particular, the HoloLens was chosen for our application since it provides significant benefits over other commercial HMD from human factors and ergonomics standpoints [27] and integrates important functionalities for an immersive and interactive simulation experience. In fact, the HoloLens offers head tracking, hand gesture controls, and voice commands and enables binaural audio to simulate effects such as spatial sound within the user environment. Additionally, HoloLens has no physical tethering constrains that can limit the movements/gestures of the user during the simulation of the surgical tasks.

A recent literature study on the evaluation of OST-HMD suitability for mixed-reality surgical intervention [28] shows that Microsoft HoloLens outperforms other currently available OST HMDs (Epson Moverio BT-200, ODG R-7), in terms of contrast perception, task load, and frame rate. The same study shows that the integration of indoor localization and tracking functionalities, enabled by HoloLens environmental understanding sensors, provides significantly less system lag in a relatively motionless scenario.

For all these reasons, HoloLens can be considered a good candidate for the implementation of mixed-reality open surgery simulators. However, some well-known technical issues of HDMs have to be considered, such as a small overlay field of view (FOV); the vergence-accommodation conflict (VAC) [29]; the perceptual issues, intrinsic to standard optical see-through HMDs, due to mismatched accommodation between the virtual content and the real-world scene [30]; and the difficulties of OST systems in handling occlusion between the real and virtual contents [26].

The overlay FOV can be defined as “the region of the field of view where graphical information and real information are superimposed” [26] which, in the HoloLens, is about 35°.

**Figure 1:**
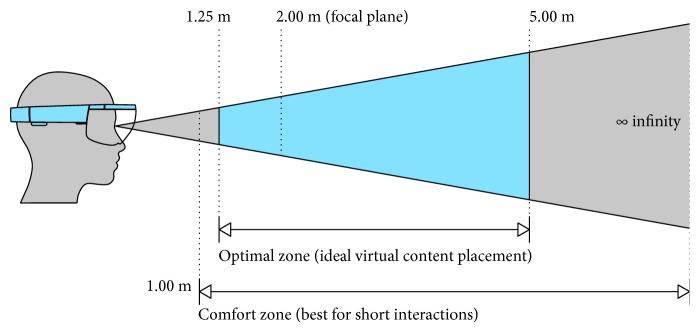
Optimal and comfort zones for placing virtual content as declared by Microsoft for HoloLens mixed-reality applications. Discomfort from the vergence-accommodation conflict can be avoided or minimized by keeping content that users converge to as close to 2.0 m as possible. When the content cannot be placed near 2.0 m, the discomfort can be reduced by keeping the virtual content positioning stable over the time.

As for the vergence-accommodation conflict, users wearing HoloLens are forced to accommodate their eyes to a fixed focal distance of approximately 2.0 m (Figure 1) to maintain a clear image of the virtual content, while the depth of the virtual objects (and hence the binocular disparity) varies depending on the application. This results in conflicting information within the vergence-accommodation feedback loops causing visual discomfort [30].

Moreover, the focal distance of each physical object in the real world depends on its relative distance from the user: if the distance gap between the display focal plane and real-world objects is beyond the human eye deep of field, the user cannot keep in focus both the virtual and real content at the same time [20].

The discomfort due to the vergence-accommodation conflict can be reduced by keeping the virtual content positioning stable over the time [31, 32]. However, the mismatch between the focal distances of real and virtual objects, together with the difficulties in handling the occlusions of overlapping objects, can affect the accuracy of the rendered depth [26].

For this reason, quantitative and qualitative tests were performed to evaluate if the perceived positioning accuracy matches the requirements of the target application. Moreover, qualitative tests were also performed to evaluate the visual discomfort and the usability of the proposed HDM for our specific scenario: orthopaedic open surgery simulation.

### 2.2. Design and Implementation of the Simulator Components: The Virtual Content

**Figure 2:**
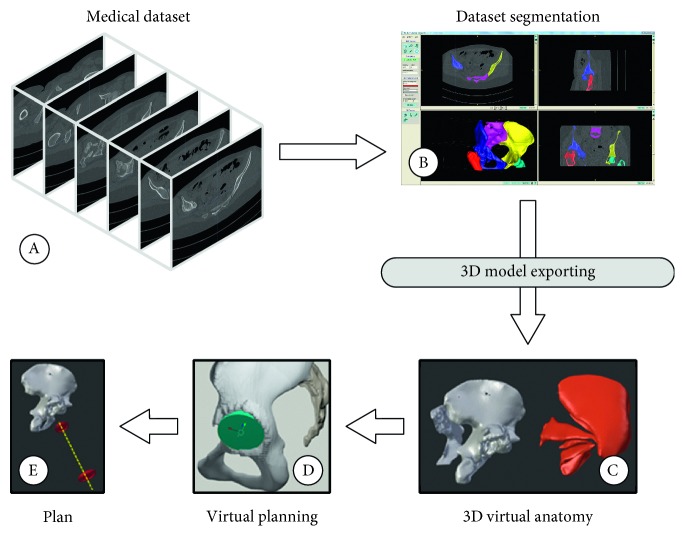
Schematic representation of the steps involved in the generation of the simulator virtual content: (A) the medical dataset of the patient; (B) the segmentation process using ITK-SNAP and the EndoCAS Segmentation Pipeline; (C) the 3D virtual anatomy generated by exporting the 3D models; (D) the virtual planning including the positioning and sizing of the acetabular component; and (E) the final preoperative plan.

The development of the simulator starts from the segmentation and surface extraction of the anatomical organs of interest from a real CT dataset (Figure 2). The stack of medical images in DICOM format is processed using a semiautomatic tool, the EndoCAS Segmentation Pipeline [33] integrated in the open source software ITK-SNAP 1.5 [34]. Then, mesh reconstruction and optimization (artefacts removal, holes filling, simplification, and filtering) stages are performed to generate the 3D models of the patient anatomy necessary for the surgical simulation. Optimization stages are performed using the open source software MeshLab [35] and Blender [36]. The bone models included in the present version of the simulator are: hip bones, sacrum, coccyx, and femoral heads. Moreover, a model of pelvis and the principal muscles around the hip joint (such as *gluteal muscles*, *piriformis*, *inferior gemellus*, *superior gemellus*, *obturator internus*) are included to increase the anatomical knowledge of the user-trainee and form a solid basis for a complete surgical simulation system. Other key surgical structures to be added for further improving the simulation are fasciae, nerves, tendons, and blood vessels.

Finally, the virtual environment is enriched with information from a simulated planning phase with the 3D Hip Plugin [23]: a pair of viewfinders and a dotted line are added to the virtual anatomical model to show the surgeon the optimal trajectory for the reaming tool. This information, coupled with the real-time tracking of the surgical instrument, could also be used for a quantitative evaluation of the surgical performance on the basis of the deviation of the reaming tool from the optimal trajectory.

**Figure 3:**
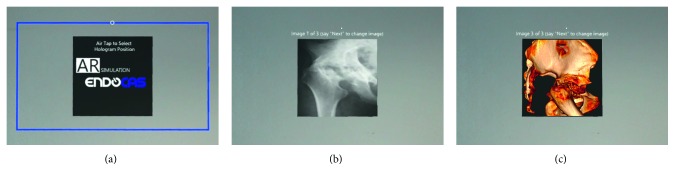
Example of AR images illustrating the medical image navigation: (a) first image presented at the beginning of the application and (b, c) two of the medical images in the collection that the user can visualize. The Air Tap gesture is used to anchor the position of medical image navigator in the physical space, whereas the voice command “Next” is used to switch the radiological images.

Moreover, a selection of radiological images (a hip radiograph, a CT slice, a CT volumetric rendering) (Figure 3) is added to the virtual content enriching the digital information available to the learner during the simulation.

### 2.3. Design and Implementation of the Simulator Components: The Physical Components

**Figure 4:**
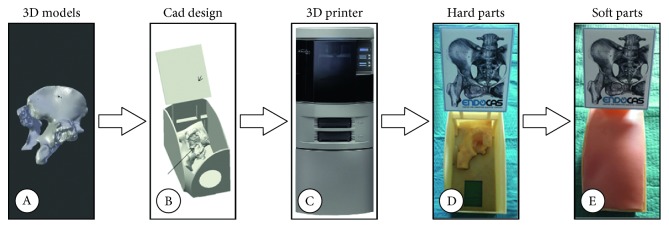
Schematic representation of the steps involved in the development and manufacturing of the physical components of the hybrid simulator: (A) the 3D model of the bones as generated from the CT dataset of the patient; (B) the CAD design for 3D printing, including the acetabulum and the support for the Image Target; (C) the 3D printer Dimension Elite; (D) and (E) the hard and soft components (respectively) of the hybrid simulator, including the Vuforia Image Target placed on top of an *ad hoc* support.

The development of the physical simulator starts from the CAD design (Figure 4). 3D virtual models are imported in the Creo Parametric 3D Modelling software, and each physical component is designed, including a support for the registration target (an Image Target as described in the following section). This support is rigidly anchored to the bone synthetic replica to guarantee a precise registration of the virtual content to the real scene.

A 3D printer (Dimension Elite 3D Printer) is used to turn the 3D CAD models into tangible 3D synthetic replicas made of acrylonitrile butadiene styrene (ABS). This plastic is commonly used for the manufacturing of bone replicas for orthopaedic surgery simulation since it adequately approximates the mechanical behaviour of the natural tissue [37]. Finally, silicone mixtures and polyurethane materials are used for the manufacturing of the soft parts.

The final mannequin includes a replica of the acetabulum embedded in a soft synthetic foam. Moreover, a skin-like covering is provided for an accurate simulation of palpation and surgical incision.

### 2.4. Calibration and Registration of the Virtual and Physical Content

Display-eye calibration and registration should be performed to properly align the virtual content with the real objects. The calibration procedure is necessary to model intrinsically and extrinsically the virtual viewing frustum to the user viewing volume. To perform this calibration, the Microsoft HoloLens includes an official “Calibration” app, which however does not offer a complete user-based calibration procedure, but it is designed to solely determine the interpupillary distance (IPD) [38].

The registration can be accomplished in real time by tracking the relative position and orientation of the real objects with respect to the rendering camera; this information is then used to update the corresponding transformations within the virtual world.

HoloLens includes a world-facing camera; thus, the optical detection and tracking of a target can be used for real-time registration purposes, with no need for an external tracking system. At this end, in our application, we use the detection and tracking functionalities offered by the Vuforia SDK [39].

**Figure 5:**
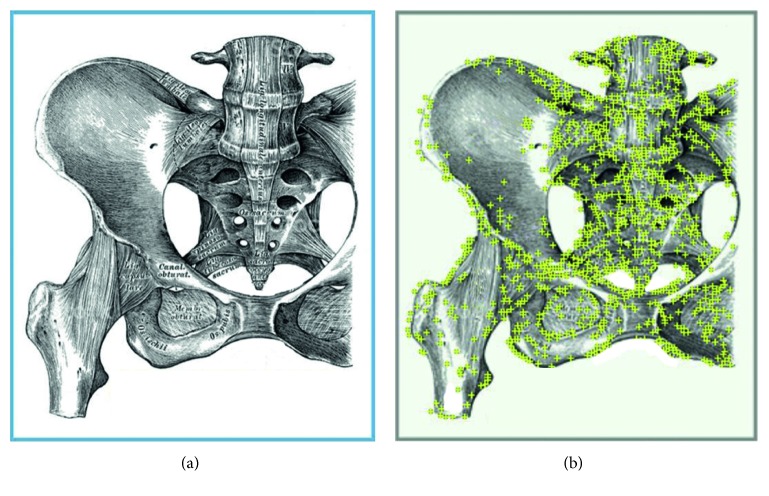
The designed Image Target that was printed with a size of 12 × 14 cm (a) and the image features detected by the Vuforia SDK (b). This Image Target obtained a 5/5-star rating: star rating defines how well an image can be detected and tracked using the Vuforia SDK, and this rating is displayed in the Target Manager and returned for each uploaded Image Target via the Vuforia web API.

More in particular, we employ an Image Target (Figure 5). Image Targets represent images that Vuforia Engine can detect and track at runtime. The Vuforia Engine detects and tracks the features that are naturally found in an image. These features, extracted from the original image, are stored in a preprocessed database, which can then be integrated in the software application. This database can then be used by Vuforia Engine for runtime comparisons. Once the Image Target is detected, Vuforia Engine will track it as long as it is at least partially visible by the camera. The fundamental attributes for an accurate tracking of an Image Target are good contrast, no repetitive patterns, and wealth of details. Moreover, for near-field applications, a physical printed Image Target should be at least 12 cm in width and of reasonable height [39]. For a more detailed definition of Vuforia Image Targets, please refer to the Vuforia SDK [39].

### 2.5. Implementation Details

From the software aspect, Unity3D (5.6.1f) was used to create the application (the HipSim app). The MixedRealityToolkit (2017.1.2), a collection of C# scripts and Unity components to develop mixed-reality applications, was utilized for the development of the surgical simulator. This toolkit allows the user to interact with the virtual content by means of head movements (Gaze), gestures (Air Tap, Bloom, etc.), and voice commands (via Cortana). A virtual cursor is added to the application to indicate the head/view direction: this interaction through head movements is called Gaze. The Gaze is estimated from the position and orientation of the user's head, without considering the user's eyes direction (since the current version of HoloLens does not include any eye-tracking sensor).

A Fitbox (a MixedRealityToolkit tool) is used in Unity to anchor in the physical space the virtual collection of radiological images according to the user preferences (Figure 3).

**Figure 6:**
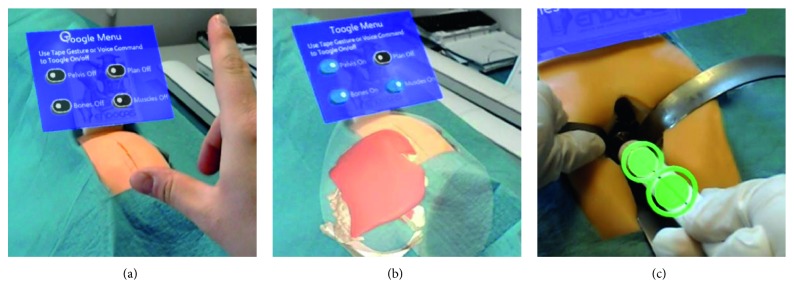
Examples of AR images captured during the simulated surgical procedure: (a) the mannequin, positioned on a surgical table and covered with a surgical drape to enhance the realism of simulation, and the virtual AR menu for the selection of the virtual anatomical components to be visualized; (b) the surgeon can visualize in AR mode the virtual anatomy before performing the surgical incision; (c) with the help of the virtual viewfinder, the surgeon can orient the surgical instrument, so that the acetabulum reaming can proceed in the direction of the planned implant.

A virtual menu with multiple toggle buttons has been implemented to select the virtual components (pelvis, bones, and muscles; preoperative plan) to be visualized during each surgical task.  Figure 6 shows examples of AR images captured by the HoloLens word-facing camera during a surgical simulation trial.

Operating room ambient sound, including voices of surgical staff and sounds of medical equipment, has been included in the HipSim app to improve the realism and immersion of the surgical simulation.

### 2.6. Quantitative Study

Quantitative tests were performed to estimate the accuracy of the system by evaluating the perceived position of AR targets.

**Figure 7:**
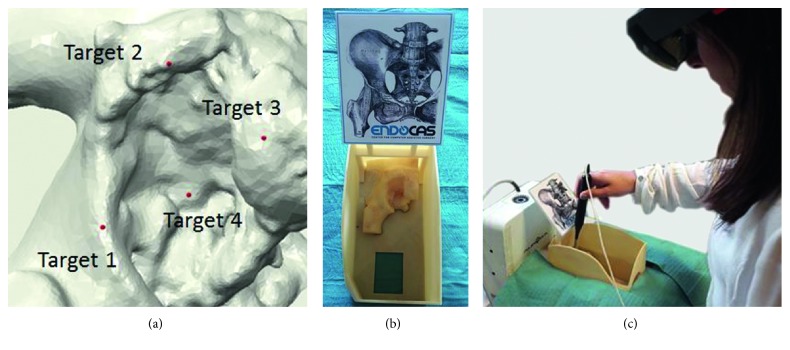
Experimental setup for the quantitative study: (a) planned position of targets (red points) in the CAD environment; (b) mannequin used for the test with the Vuforia Image Target; (c) a user wearing the HoloLens during a targeting task and using the Aurora digitizer to point at the perceived position of one target.

Five (5) subjects (gender: 2 males, 3 females, 0 nonbinary; years of age: 24 min, 32 mean, 39 max, 6 STD) with 10/10 vision were recruited to participate in this study. The HoloLens were used to present four (4) virtual targets consisting of red spheres (0.5 mm radius) virtually located on the *acetabulum* surface (Figure 7(a)). Targets were designed in the CAD environment and their 3D positions were acquired in the virtual environment reference frame.

Figure 7 shows the experimental setup consisting of:

- the Microsoft HoloLens HMD;
- the rigid components of the mannequin, without the synthetic soft tissue;
- the Vuforia SDK Image Target for tracking and registration;
- the NDI Aurora electromagnetic tracking system (V2 System); and
- the NDI Aurora calibrated 6 degrees of freedom (DOF) digitizer.

The mannequin and the Aurora EM emitter were fixed in a stable position to avoid relative movement during the targeting trials.

The rigid transformation ^**A**^**T**_**V**_ between the Aurora reference system and virtual environment reference frame was derived with a point-based registration algorithm: the positions of three landmarks (three corners of the simulator) were acquired in the CAD environment; the positions of the same landmarks were then acquired in the Aurora reference system with the digitizer; and then the transformation was derived with a least-squares error minimization algorithm [40]. Finally, the root mean squared registration error (RMSE) and the maximum registration error (MR) were computed and saved.

The official HoloLens app was used to calibrate the HMD for each user before the targeting session. The tracking and registration functionalities supported by the Vuforia SDK were used for the real-time registration of the virtual targets and the real mannequin.

The subjects were asked to use the digitizer to point at the perceived position of the four (4) virtual targets displayed through the HMD (Figure 7). Each target was acquired 3 times by each user, for a total of 12 targeting trials per person (60 in total). Target positions, acquired in the Aurora reference frame, were then expressed in the virtual environment reference frame by means of the ^**A**^**T**_**V**_ rigid transformation.

Targeting accuracy was measured as the average Euclidean distance between the perceived (digitized) position and the actual position of each target. The maximum and minimum error (Euclidean distance), as well as the standard deviation, were also calculated for each target.

### 2.7. Qualitative Study

Twenty (20) subjects with 10/10 or corrected (lenses) to 10/10 vision were recruited from technical employees (engineers) and personnel with medical background (medical students, orthopaedic resident surgeons, orthopaedic surgeons) of the University of Pisa (see Table 1 for detailed demographics).

The qualitative study includes: subjective workload assessments with the NASA Task Load Index (NASA-TLX) Questionnaire and a Likert Questionnaire to evaluate visual and audio perception, and interaction and ergonomics issues. NASA-TLX is a multidimensional rating procedure that provides an overall workload score, between 0 and 100, based on a weighted average of ratings on six subscales [41]:

- mental demands (“How mentally demanding was the task?”),
- physical demands (“How physically demanding was the task?”),
- temporal demands (“How hurried or rushed was the pace of the task?”),
- own performance (“How successful were you in performing the task?”),
- effort (“How hard did you have to work to achieve your level of performance?”), and
- frustration (“How insecure, discouraged, irritated, stressed, and annoyed were you?”).

NASA-TLX Questionnaire was administrated to identify the primary source of workload during the execution of the proposed AR-based simulation and to investigate workload levels of users with differing characteristics (“Profession/Position Held,” “Experience with AR” etc.).

The Likert Questionnaire, which is reported in Table 2, comprises 14 items, each evaluated using a 5-points Likert scale (from 1 = strongly disagree, to 5 = strongly agree).

**Figure 8:**
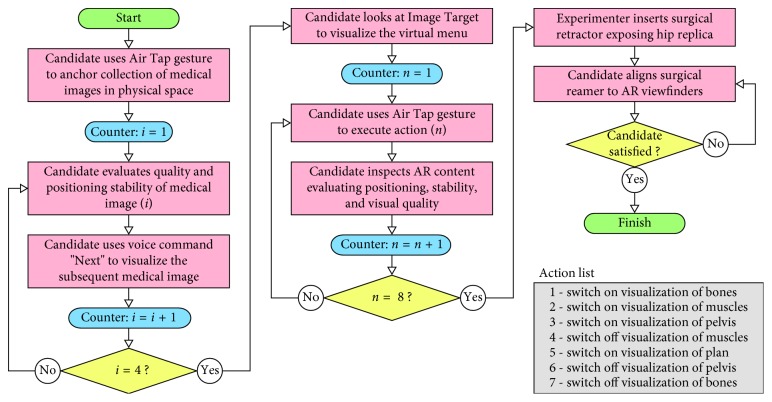
Flowchart of the experiment using the HipSim app.

The experimental setup is depicted in Figure 6. The mannequin was positioned on a fixed height surgical table. The study protocol for each participant included the following steps:

1. The participant fills out a Consent Form and a Demographic Form (Table 1) including information about his/her previous experience with AR and HoloLens.
2. The subject calibrates the HoloLens using the Calibration app (by Microsoft).
3. The subject learns how to interact with HoloLens by means of head movements, gestures, and voice commands, using the Learn Gestures app (by Microsoft).
4. The subject fills out the NASA-TLX Questionnaire (part 1, weights form).
5. The HipSim app is launched and the subject has to perform a series of tasks (Figure 8).
6. The subject fills out the NASA-TLX Questionnaire (part 2, rating form).
7. The subject fills out the Likert Questionnaire.
8. The total time of the study was recorded for every participant.

Statistical analysis of data was performed using the SPSS® Statistics Base 19 software.

Results of the NASA-TLX Questionnaire are summarized in terms of means and standard deviation. Data were processed using the analysis of variance (ANOVA) to examine possible relationships between individual characteristics and workload.

As for the Likert Questionnaire, the central tendencies of responses to a single Likert item were summarized by using median, with dispersion measured by interquartile range. The Mann–Whitney *U* test and Kruskal–Wallis test were used to understand whether the answering tendencies (with respect to each Likert item) differ based on “Profession/Position Held” and “Experience with AR”/“Experience with HoloLens”. A *p* value <0.05 was considered statistically significant.

## 3. Results

### 3.1. Quantitative Evaluation Results

The obtained RMSE and MR are, respectively, 0.6 mm and 0.8 mm. Table 2 reports the accuracy obtained for each target, as well as the maximum error, minimum error and the standard deviation. The maximum error is compatible with values declared by HoloLens developers: Klein G. reported [42] a maximum static registration error <10 mrad, which results in an error of about 5 mm at a distance of 50 cm from the user (the approximate working distance in our setup).

### 3.2. Qualitative Evaluation Results

The average time for the completion of the study was 40 minutes.

**Figure 9:**
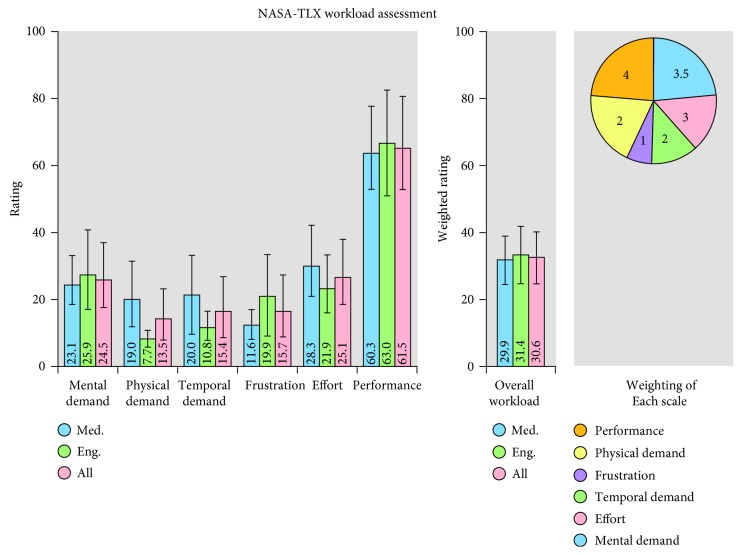
The bar charts show the mean rating with the standard deviation of each subscale and the overall weighted workload for personnel with medical background, engineers, and all participants. The pie chart shows the averaged (across all participants) weighting for each subscale (the total weighting is 15).

Figure 9 shows the results of the subjective workload scores from the NASA-TLX Questionnaire. No statistically significant differences were found between personnel with medical background and engineers (Mental Demand *p*=0.741; Physical Demand *p*=0.079; Temporal Demand *p*=0.246; Frustration Demand *p*=0.297; Effort *p*=0.445; Performance Evaluation *p*=0.826; Overall Workload *p*=0.825). Moreover, no statistically significant differences were found between groups with different experience with AR (Mental Demand *p*=0.418; Physical Demand *p*=0.539; Temporal Demand *p*=0.524; Frustration Demand *p*=0.912; Effort *p*=0.218; Performance Evaluation *p*=0.709; Overall Workload *p*=0.931); and HoloLens (Mental Demand *p*=0.419; Physical Demand *p*=0.800; Temporal Demand *p*=0.718; Frustration Demand *p*=0.831; Effort *p*=0.530; Performance Evaluation *p*=0.704; Overall Workload *p*=0.905).

The overall workload obtained (30.65) can be considered low giving that the average overall score observed in the literature for medical task is 50.60 (min 9.00; max 77.35) and for computer activities is 54.00 (min 7.46; max 78.00) [43]. Performance induced the highest workload indicating the overall satisfaction with self-assessed performance.

Table 3 summarizes the results of the Likert Questionnaire. Results show no statistically significant differences in answering tendencies between engineers and clinicians with an exception for the postural discomfort during the application and the ease of aligning the surgical instrument to the AR viewfinders.

As for the postural discomfort, clinicians expressed a neutral opinion (median 3), while engineers agreed that they did not experience postural discomfort (median 4). Moreover, clinician also expressed a neutral opinion (median 3) regarding the ease of aligning the surgical instrument, while engineers strongly agreed that this task is easy (median 5).

Overall, participants agreed/strongly agreed that the virtual content is correctly aligned to the real objects (median 5), it is easy to perceive the spatial relationships between real and virtual objects (median 5), they did not notice motion of virtual content (median 4), they did not notice latency (median 4), they did not notice jitter (median 4), they did not experience double vision (median 5), they did not notice colour separation (median 5), the field of view is adequate for the application (median 4), the spatial sounds make the experience more immersive (median 4.5), the gesture interaction is easy and intuitive (median 5), and the voice interaction is easy and intuitive (median 4.5). The overall median opinion regarding the experience of visual fatigue is neutral (median 3.5).

## 4. Conclusions

As suggested by a recent literature review on orthopaedic surgery simulation [11], *“an ideal simulator should be multimodal, combining haptic, visual and audio technology to create an immersive training environment.”* In this work, we present an innovative multimodal simulation tool, which takes advantage from patient-specific modelling to improve the realism of the simulated surgical case; rapid prototyping for the manufacturing of synthetic models, which guarantees a realistic haptic feedback; AR to enrich the simulated scenario and guide the learner during the surgical procedure; and HoloLens functionalities for an interactive and immersive simulation experience.

Results of quantitative and qualitative study encourage the usage of HoloLens technology for the implementation of a hybrid simulator for orthopaedic open surgery. The perceived positioning accuracy matches the requirements of the target application. Moreover, the perceived overall workload can be considered low, and subjects participating in this study expressed satisfaction with self-assessed performance. A positive feedback was obtained on visual and audio perception, and gesture and voice interaction independently of the level of previous experience with AR and HoloLens, and education backgrounds (medical or technical). As regards postural discomfort during the application and the experience visual fatigue, obtained results show a nonnegative opinion for a simulation experience with duration of 40 minutes (enough for the specific purposes). A more prolonged usage could negatively impact the comfort because of an increase of the visual fatigue. An optimal design of the simulation tasks and the simulation setup (time for each task, height of the surgical table, distance of user interaction) are required to minimize the user discomfort, so that the virtual content appears in the optimal/comfort zone for most of the time of the simulation period, and the head tilt is sustainable. Moreover, attention should be paid to the design of AR viewfinders (optimal shape, colour, transparency level) to ease the alignment task, which is already impaired by the focus rivalry between the physical and virtual content.

Hip arthroplasty, a surgical procedure which could take great advantage from simulation, was selected as a benchmark for this study. Primary and revision total HA indeed were ranked third and fourth among the orthopaedic interventions accounted for the greatest share of adverse events and excess hospital stay [44] and, as showed by several studies [45, 46], the risk of complications after HA is strongly related to the surgeon's case volume. In this context, surgical simulation could play a pivotal role, offering novices an opportunity to practice skills outside the operating theatre, in a safe controlled environment.

Future work will include Face Validity, Content Validity, and Construct Validity for a complete assessment of the proposed simulator for this specific orthopaedic intervention. Additionally, in the future, our system could integrate novel haptic equipment and able to simulate high-magnitude force feedback. However, in this case, the usage of haptic interfaces will be limited to the simulation of the reamer-bone interactions, whereas the direct interactions between the surgeon hands and the soft tissue will be still simulated using the current synthetic mannequin.

## Figures and Tables

**Table 1:** Demographics of participants in the qualitative evaluation study.

*Profession/Position Held* (engineers; med. staff: students, orthop. residents, and orthop. surgeons)	10; 10 (6, 1, 3)
*Gender* (male, female, nonbinary)	13, 7, 0
*Age* (min, max, mean, STD)	23, 48, 32, 7
*Handedness* (left, right, ambidextrous)	2, 18, 0
*Vision* (10/10 naked eyes, corrected to 10/10 with lenses)	10, 10
*Experience with AR* (none, limited, familiar, experienced)	8, 5, 5, 2
*Experience with HoloLens* (none, limited, familiar, experienced)	16, 3, 1, 0
*Colour Blindness* (no, yes)	20, 0
*English Reading* (none, limited, familiar, experienced)	0, 0, 12, 8
*English Speaking* (none, limited, familiar, experienced)	0, 2, 11, 6

**Table 2:** Spatial accuracy evaluation.

	Accuracy (mean error)	Max. error	Min. error	STD
Target 1	2.1	4.4	1.0	1.1
Target 2	1.7	3.3	0.9	0.8
Target 3	1.7	3.3	0.7	0.7
Target 4	2.5	5.2	0.8	1.4
Total	2.0	5.2	0.7	1.1

**Table 3:** Qualitative evaluation using a 5-point Likert questionnaire. Central tendency summarized using median with dispersion measured by interquartile range (25°∼75°).

	Item	Questionnaire items	Median (25°∼75°)			*P* value (Eng. vs Clin.)
			Engineers	Clinicians	All	
Visual and audio perception	A	The virtual content is correctly aligned to the real objects.	5 (5∼4)	4 (5∼3.75)	5 (5∼4)	0.280
	B	It is easy to perceive the spatial relationships between real and virtual objects.	5 (5∼4)	4.5 (5∼4)	5 (5∼4)	0.739
	C	I did not notice motion of virtual content.	4 (5∼4)	4 (5∼3.75)	4 (5∼4)	0.436
	D	I did not notice latency (lag, delay) between virtual content and objects real.	4.5 (5∼4)	4 (5∼4)	4 (5∼4)	0.353
	E	I did not notice jitter (high-frequency shaking of the virtual content).	4 (5∼2.75)	4 (4.75∼3.75)	4 (5∼3)	0.912
	F	I did not experience double vision.	4.5 (5∼4)	5 (5∼4)	5 (5∼4)	0.481
	G	I did not notice colour separation.	5 (5∼3.75)	5 (5∼4.75)	5 (5∼4)	0.393
	H	The field of view (FOV) is adequate for the application.	4 (4.25∼2.75)	3.5 (4∼2.0)	4 (4∼2.25)	0.579
	I	Spatial sounds make the experience more immersive.	4 (5∼4)	5 (5∼3.75)	4.5 (5∼4)	0.796

Interaction and ergonomics	J	I did not experience postural discomfort during the application.	4 (4.25∼3.75)	3 (4 ∼ 2)	4 (4∼2.25)	**0.029**
	K	I did not experience visual fatigue (eyestrain, dried mucus or tears around the eyelids, discomfort when the eyes are open, hot eyes, and headaches).	4 (4.25∼2.75)	2.5 (4.25∼2)	3.5 (4∼2)	0.393
	L	Gesture interaction is easy and intuitive.	4.5 (5∼4)	5 (5∼4)	5 (5∼4)	0.631
	M	Voice interaction is easy and intuitive.	4 (5∼4)	5 (5∼4)	4.5 (5∼4)	0.481
	N	It is easy to aligning the surgical instrument to the AR viewfinders.	5 (5∼4)	3 (4∼2)	4 (5∼3)	**0.023**

No statistically significant differences were found between groups with different experience with AR (Item A *p*=0.126; Item B *p*=0.219; Item C *p*=0.789; Item D *p*=0.653; Item E *p*=0.590; Item F *p*=0.085; Item G *p*=0.204; Item H *p*=0.466; Item I *p*=0.196; Item J *p*=0.204; Item K *p*=0.246; Item L *p*=0.469; Item M *p*=0.284; Item N *p*=0.193) and HoloLens (Item A *p*=0.606; Item B *p*=0.662; Item C *p*=0.772; Item D *p*=0.326; Item E *p*=0.986; Item F *p*=0.986; Item G *p*=0.772; Item H *p*=0.499; Item I *p*=0.364, item J *p*=0.470; Item K *p*=0.508; Item L *p*=0.739; Item M *p*=0.187; Item N *p*=0.760).

## Data Availability

The data used to support the findings of this study are available from the corresponding author upon request.
